# Non-steroidal Anti-inflammatory Drug-induced Acute Pancreatitis: A Case Report

**DOI:** 10.7759/cureus.5926

**Published:** 2019-10-16

**Authors:** Jonathan Vincent Reyes, Bhavin M Patel, Fahad Malik, Manuel O Gonzalez

**Affiliations:** 1 Internal Medicine, Icahn School of Medicine at Mount Sinai, Elmhurst Hospital Center, Elmhurst, USA; 2 Gastroenterology, Newyork-Presbyterian Hospital, Brooklyn, USA; 3 Internal Medicine, University of Alabama at Birmingham, Montgomery, USA; 4 Gastroenterology, Richmond University Medical Center, Staten Island, USA

**Keywords:** pancreas, pancreatitis, ibuprofen, gastrointestinal side effects, side effects, non-steroidal anti-inflammatory drugs (nsaids)

## Abstract

Ibuprofen-induced acute pancreatitis, a diagnosis secondary to the use of non-steroidal anti-inflammatory drugs (NSAIDs), is an extremely rare occurrence. Common culprits, such as gallbladder obstruction, alcohol consumption, infection, direct trauma, and medication (i.e. NSAIDs), can be attributable to the majority of cases reported. This case report describes a patient with acute pancreatitis that developed due to a three-week course of daily ibuprofen use for chronic shoulder pain. Alternative causes of acute pancreatitis were excluded through the patient’s clinical history, laboratory findings, and diagnostic imaging. Although a rare risk factor, our aim is to further demonstrate that patients with chronic NSAIDs use can develop these complications and this should be considered among the differential diagnoses.

## Introduction

Drug-induced acute pancreatitis was first reported in the 1950s and the list of potential drug associations with acute pancreatitis has increased every year [1]. Accurate diagnosis is an important step to prevent recurrence and decrease mortality due to acute pancreatitis. Further, 0.1% to 2% of acute pancreatitis is secondary to medications while the most common causes are gallstones in about 35%-40% of cases and alcohol use in about 30% of cases [2-3]. As the stereotypical causes of acute pancreatitis remain common, it is important for physicians to consider other predisposing factors when considering acute pancreatitis in the differential diagnoses. Such characteristics include being female, immunosuppressed patients, transplant recipients, and patients taking immunomodulators [4]. These risk factors also increase the likelihood of drug-induced pancreatitis. Numerous drug classes have been connected to acute pancreatitis through published reports. The following medications represent an extensive summary of notable causes: angiotensin-converting-enzyme inhibitors, furosemide, calcium channel blockers, thiazides, methyldopa, didanosine, stavudine, non-steroidal anti-inflammatory drugs (NSAIDs), lamivudine, nelfinavir, ritonavir, valproic acid, clozapine, azathioprine, 6-mercaptopurine, ketoconazole, metronidazole, isoniazid, pentamidine, tetracycline, Interferon alpha-2b, metformin, sitagliptin, linagliptin, vildagliptin, saxagliptin, liraglutide, exenatide, estrogen, octreotide, and corticosteroids [2-4]. Most NSAIDs have been associated with causing acute pancreatitis but the most notorious of them all is sulindac [5]. Upon an extensive literature review, we found two other cases of ibuprofen-induced pancreatitis [6-7].

## Case presentation

Our patient was a 44-year-old female with a past medical history significant for hyperlipidemia, cholelithiasis status post cholecystectomy, and acute pancreatitis secondary to choledocholithiasis. She presented with a two-week history of intermittent abdominal pain. This pain was described as “burning” in nature, radiated to her back and was 8/10 in intensity. It was associated with nausea, vomiting, and diarrhea but not associated with eating. The patient reported that she did not use any palliative measures for the pain but indicated that she had been taking 800 mg Ibuprofen every eight hours for the past three weeks for an acute flare-up of chronic right shoulder pain. The patient denied recent travel, sick contacts at home, or any change in eating habits. She also denied Ibuprofen use during the last episode of acute pancreatitis in 2010. The patient was a non-tobacco smoker, non-alcohol drinker, and did not use any illicit drugs.

Upon initial evaluation in the emergency department, the patient was found lying on the stretcher, in a moderate amount of pain but agreeable to questioning and examination. Initial vital recordings were as follows: temperature 98.6 Fahrenheit, heart rate 66 beats per minute, respiratory rate 18 breaths per minute, blood pressure 99/60 millimeters of mercury, pulse oximetry of 100% on room air, pain scale 8/10, and a body mass index (BMI) of 26.0. The neurological exam consisted of cranial nerves 2-12 intact, no focal deficits observed. The cardiovascular exam showed an electrocardiogram with normal sinus rhythm and no murmurs, rubs, or gallops were heard. The chest examination was with no visible deformities or evidence of trauma, and the lungs were clear to auscultation bilaterally, with breath sounds heard to the lung bases. The abdominal exam revealed positive bowel sounds in all four quadrants and increased epigastric tenderness was elicited while the patient affirmed symptoms of radiating pain to the back with nausea and vomiting. Contributing laboratory studies included elevated amylase to 739 U/L (normal range 0 - 158 U/L), elevated lipase to 12,041 U/L (normal range 0-160 U/L), and elevated triglycerides to 238 mg/dL (normal range 0 - 115 mg/dL). The abdominal computed tomography (CT) confirmed the suspected diagnoses, indicating acute pancreatitis with mild to moderate amount of pancreatic ascites tracking into the pelvis and without focal fluid collection to suggest phlegmon or pseudocyst. Magnetic resonance cholangiopancreatography (MRCP) was also conducted and revealed evidence of pancreatitis with diffuse surrounding edema and ascites, confirming the absence of stones in the common bile duct, with a diameter of 8 mm. Imaging for the scans mentioned above was unfortunately lost in a system-wide update. Initially, the patient was treated with ondansetron 4 mg intravenous (IV), famotidine 20 mg IV, morphine 4 mg IV, and normal saline IV bolus x two bags.

Differential diagnoses

The etiology of this patient’s acute pancreatitis was in question, primarily due to the fact that the patient had a significant past medical history of choledocholithiasis status post cholecystectomy, ruling out one of the primary causes of acute pancreatitis. She also presented with mildly elevated triglyceride levels, another common cause of acute pancreatitis, but the levels of triglycerides were unimpressive, as more than 1000 mg/dL is generally seen as an identifiable risk factor. The patient’s social risk factors were also negative for tobacco or ethanol use. Based on the patient’s non-contributory past medical history, idiopathic and ibuprofen-induced etiologies were considered. Differentiating the cause, however, did not influence the management of this patient with the exception that the patient ceased ibuprofen use at this time. For more information regarding potential causes of acute pancreatitis, see Table 1.

**Table 1:** Differential diagnosis for acute pancreatitis CBD: Common Bile Duct, MRCP: Magnetic Resonance Cholangiopancreatography; NSAIDs: Non-Steroidal Anti-Inflammatory Drugs

Etiology	Pertinent Positives	Pertinent Negatives
Retained gallstone in CBD		MRCP confirmed no evidence of retained stones in CBD; Negative for dilation of CBD >9mm; History of cholecystectomy
Hypertriglyceridemia	Evidence of mild hypertriglyceridemia	Only mildly elevated triglycerides, lacking the power of hypertriglyceridemia typically seen in acute pancreatitis
Idiopathic	Diagnosis of exclusion	MRCP confirmed no evidence of retained stones in CBD; Negative for dilation of CBD >9mm; History of cholecystectomy; Mildly elevated triglycerides; No ethanol use
Ibuprofen - induced	Patient taking high levels of NSAIDs daily	MRCP confirmed no evidence of retained stones in CBD; Negative for dilation of CBD >9mm; History of cholecystectomy; Mildly elevated triglycerides; No ethanol use

Treatment

The treatment plan for the patient included intravenous hydration and maintenance of electrolyte neutrality with IV lactated Ringers at 100 ml every five hours until the patient was advanced to a clear liquid diet and eventual regular diet. The patient’s labs and vitals were trended daily, including lipid panels, amylase, lipase, liver function tests (LFTs), complete blood count (CBC), and comprehensive metabolic panel (CMP). The patient was monitored for acute changes in respiratory status. From a gastrointestinal perspective, the pain was managed with morphine 2 mg every four hours, as needed for the subjective pain scale at 5+/10 and ondansetron 4 mg every six hours as needed was given for antiemesis. The patient did not receive antibiotics during her hospital course. She received deep vein thrombosis (DVT) prophylaxis with sequential compression device stockings.

Outcome and follow-up

The patient was discharged three days after inpatient admission. She was managed conservatively and symptomatically with the cessation of all NSAIDs. The patient clinically improved on a daily basis.

The patient was advised to follow-up with a gastroenterologist in the outpatient setting for repeat imaging of the pancreas and was advised to seek alternative solutions for pain management. She was also advised to follow-up with an orthopedic specialist for her chronic shoulder pain.

## Discussion

As mentioned earlier, the incidence of drug-induced pancreatitis (DIP) is low and generally includes β-Hydroxy β-methyl-glutaryl-coenzyme (HMG-CoA) reductase inhibitors, angiotensin-converting-enzyme (ACE) inhibitors, oral contraceptives or hormone replacement therapy, diuretics, highly active antiretroviral therapy (HAART) therapy, and valproic acid as some of the more common offending agents. One specific case report has been regarded as “decisive” in determining whether a suspected drug is causal for acute pancreatitis [2]. The report is a well-documented episode of recurrent acute pancreatitis, which was induced by rechallenge with the suspected drug. The criterion was proposed by Mallory and Kern, as given below:

1. Pancreatitis develops during treatment with a drug, resolves upon discontinuing the drug, and returns upon the readministration of the drug.

2. Adequate criteria for the diagnosis of acute pancreatitis are present both for the initial episode of acute pancreatitis and the episode following the rechallenge.

3. Other likely causes of pancreatitis are not present [8].

Experts created a classification system of the likelihood that specific drugs would be associated with acute pancreatitis, placing drugs into the following categories: definite, probable, and questionable/possible [1]. In a review by Badalov et al., class I drugs, or those with the strongest evidence for a causal role in acute pancreatitis, were classified if there was at least one published positive rechallenge case report. Class I was further stratified into classes Ia and Ib, in which Ia included causes such as alcohol, hypertriglyceridemia, gallstones, and other drugs that were excluded and Ib for all other causes not ruled out. Classes II, III, and IV were identified but do not require documentation of a positive rechallenge test [7].

The diagnosis of DIP requires an initial diagnosis of acute pancreatitis as per current recommended guidelines, specifically two of the following three criteria must be met: (1) serum amylase and lipase elevations at least three times the upper limit of normal, (2) epigastric abdominal pain often radiating to the back that is not aggravated by movement, respiration, or coughing that is more severe in the supine position, and (3) typical radiological features [4]. In addition, causality is based on ruling out common etiologies with comprehensive history taking, other imaging modalities, such as CT or MRCP, laboratory indicators, and serum ethanol toxicity levels.

The mechanisms of these drugs are not well-known and extracted from theories outlined in case reports, case-control studies, animal studies, and other experimental data. It is noted in one review that potential mechanisms may include pancreatic duct constriction, direct cytotoxic and metabolic effects, accumulation of a toxic metabolite intermediary, and hypersensitivity reactions [2]. One case report of ibuprofen-induced pancreatitis suggests a possible mechanism related to a reduction in systemic glutathione caused by decreased superoxide dismutase activity, which subsequently prevents an appropriate response against oxidative stresses [3-4]. Another potential theory for the mechanism of pancreatitis secondary to NSAIDs is due to the destabilizing effect of NSAIDs on the pancreatic cell membrane by affecting prostaglandins [9]. Treatment for DIP is the discontinuation of the offending agent, and resolution of acute pancreatitis after doing so increases the likelihood of the suspected diagnosis.

While implicated as a cause of pancreatitis, it is important to be aware that NSAIDs have also been studied to have a protective effect on the pancreas in very specific circumstances [10]. Research has been done studying the efficacy of NSAIDs after an ERCP procedure to prevent pancreatitis. The majority of studies reported the method of NSAID delivery through rectal administration [11-12]. This discrepancy presents an opportunity for future research to explore this paradox.

## Conclusions

In this case of suspected diagnosis of Ibuprofen-induced acute pancreatitis, we did not perform a rechallenge test on the patient to confirm the suspected diagnosis; however, the patient demonstrated no other contributing factors that could be linked to the condition. There are few reasons to rechallenge a medication to determine whether or not it is the culprit for pancreatitis. The patient endorsed no medication use besides ibuprofen. Common etiologies, such as gallstones, ethanol use, and hypertriglyceridemia, were all excluded. We conclude Ibuprofen to be the cause. While NSAIDs are known to cause pancreatitis, albeit rarely, this case report will serve as evidence for this rare complication. After common causes of acute pancreatitis have been ruled out, physicians should consider NSAID-induced pancreatitis as an additional differential diagnosis.
